# Pet dog facial expression recognition based on convolutional neural network and improved whale optimization algorithm

**DOI:** 10.1038/s41598-023-30442-0

**Published:** 2023-02-27

**Authors:** Yan Mao, Yaqian Liu

**Affiliations:** 1 College of Information Engineering, Tongji University, Shanghai, China; 2 Present Address: College of Animal Science and Technology, Shandong Agricultural University, Tai’an, Shandong China

**Keywords:** Computational science, Computer science, Information technology, Software, Statistics

## Abstract

Pet dogs are our good friends. Realizing the dog’s emotions through the dog's facial expressions is beneficial to the harmonious coexistence between human beings and pet dogs. This paper describes a study on dog facial expression recognition using convolutional neural network (CNN), which is a representative algorithm model of deep learning. Parameter settings have a profound impact on the performance of a CNN model, improper parameter setting will make the model exposes several shortcomings, such as slow learning speed, easy to fall into local optimal solution, etc. In response to these shortcomings and improve the accuracy of recognition, a novel CNN model based on the improved whale optimization algorithm (IWOA) called IWOA–CNN is applied to complete this recognition task. Unlike human face recognition, a dedicated face detector in Dlib toolkit is utilized to recognize the facial region, and the captured facial images are augmented to build an expression dataset. The random dropout layer and L2 regularization are introduced into the network to reduce the number of transmission parameters of network and avoid over fitting. The IWOA optimizes the keep probability of the dropout layer, the parameter λ of L2 regularization and the dynamic learning rate of gradient descent optimizer. Carry out a comparative experiment of IWOA–CNN, Support Vector Machine, LeNet-5 and other classifiers for facial expression recognition, its results demonstrate that the IWOA–CNN has better recognition effect in facial expression recognition and also explain the efficiency of the swarm intelligence algorithm in dealing with model parameter optimization.

## Introduction

Expression is one of the important ways for creatures in nature to express emotions. As early as 1968, Albert Mehrabian, a famous psychologist, pointed out that 55% of emotional information is transmitted by facial expressions. Different from human beings who are good at camouflage, animals do not hide their minds, and their expressions can better reflect real emotions. As the most accessible animals in our life, pet dogs’ expressions are meaningful study objects. Expression recognition is the main way for computers to understand expression information, and its research can be traced back to the 1970s, at that time, two American scholars Ekman and Friesen took the lead in studying the relationship between muscle movement and facial expression, and proposed a facial expression coding system. The emergence of deep learning has realized the extraction of image features in the way of autonomous learning, and has also provided a new research method for expression recognition^1,2^. Expression recognition technology is widely used, including fatigue driving detection^3^, safety monitor^4^, teaching monitoring^5^, pain identification^6^, etc.

**Figure 1:**
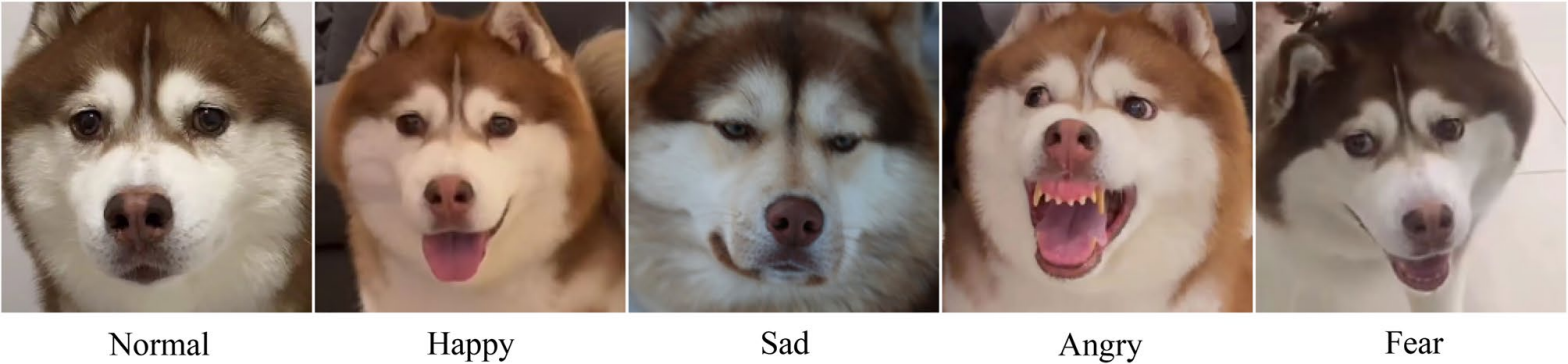
Different expressions of a pet dog.

There are already a number of expression recognition databases for experiments^7^, such as the Facial Expression Recognition 2013 (FER2013), the Extended Cohn-Kanade (CK+), and the Real-world Affective Faces Database (RAF-DB), etc. But almost all of them are collected from human faces, there is rare dataset based on animal faces, thus, getting images of dog facial expressions is also an arduous task. We collected 315 facial images of dogs in natural scenes and divided them into five different expressions, including normal, happy, sad, angry and fear. Figure 1 shows a dog with five different facial expressions. As a reason of the facial features of dogs are different from those of humans, dogs’ face detection and expression recognition may be a difficult task, the techniques and tools used to perform the task need to be chosen properly.

It is known to us that convolutional neural network (CNN)^8^ is one of the most effective model architectures for image classification. Many CNN based network frameworks have been proposed, such as AlexNet^9^ and ZFNet^10^ with fewer convolutional layers, and others with deeper layers, such as VGG^11^, Google Inception^12^ and ResNet^13^. CNN plays an important role in the research of facial expression recognition, which has helped many researchers to achieve fruitful results in the field of image recognition. Lopes et al. tried to extract facial features using preprocessing technology and input them into 5-layer CNN for facial expression recognition, achieving a recognition rate of 97.95% on CK+ dataset^14^. Li et al.^15^ proposed an attention mechanism-based CNN for facial expression recognition, and achieved 75.82% and 98.68% accuracy respectively on FER2013 and CK+ datasets. Qin et al.^16^ proposed a method combining Gabor wavelet transform and 2-channel CNN for facial expression recognition, which can achieve an accuracy of 96.81% on CK+ dataset. Li et al.^17^proposed a method combining Local Binary Patterns (LBP) and deep-CNN for celebrity face recognition, which reaches 80.35% and 99.56% accuracy on CelebA and LFW datasets respectively. The above research results indicate that in addition to selecting the correct algorithm model, facial region localization and facial feature extraction are also very important to increase the accuracy of recognition. In this article, we use the Dlib library to detect the dog's facial region, it is based on the ensemble of regression trees (ERT) algorithm^18^, and has high speed in face detection. After the face region is located, it is captured and converted into grayscale image, then input the image into a CNN model to extract its feature.

Swarm intelligence optimization algorithm (SIOA) has been proposed in recent years, including Particle Swarm Optimization (PSO), Grey Wolf Optimizer (GWO), Sine Cosine Algorithm (SCA), Whale Optimization Algorithm (WOA), Salp Swarm Algorithm (SSA), etc. It has been successfully applied to a variety of optimization problems, like image processing^19^, feature selection^20^, path planning^21^, etc. Owing to the gradient-free property of SIOA, the algorithm is superior to traditional methods in solving optimization problems, many scholars have studied its improvement methods and widely applied it to various types of problems^22,23^. Generally speaking, the process of SIOA to solve optimization problems can be divided into exploration and exploitation stages, in which the global and local search operations will be carried out respectively. The balance of these two stages will directly affect the solution accuracy and convergence speed of the algorithm. Too many exploration operations will lead to the decline of solution accuracy, while too many exploitation operations will lead to local optimality. To avoid these possible adverse results, it is significant to take effective measures to improve the original algorithm. In this article, we propose an improved Whale Optimization Algorithm (IWOA) for optimizing parameters of the CNN model, which adopts a nonlinear convergence factor, adaptive weight strategy and differential mutation strategy.

The main contributions of us in this article can be summarized as follows:

- Images of various pet dogs are collected by us, a dedicated face detector and a feature predictor in Dlib library are utilized to recognize the dog's facial region, a CNN model is used for feature extraction of the processed images.
- In order to provide the CNN model with a large amount of data required for operation, we have enhanced the captured facial images for obtaining the dataset used in the experiment, which can promote the CNN model learn the image feature better.
- We design a CNN model architecture, which includes the random dropout layer and the L2 regularization to reduce the number of transmission parameters in the layers of the network and prevent the model from over fitting.
- The original WOA is easy to trap into local optimal solution, and not perform well in exploring the global optimal solution, thereupon, we apply a nonlinear convergence factor, adaptive weight strategy and differential mutation strategy to obtain the improved algorithm called IWOA.
- The IWOA is used to optimize parameters of the CNN model, then the optimized model (IWOA-CNN) and relevant classifiers are applied to the dog expression recognition experiment and human expression recognition experiments based on several ready-made datasets. By comparing the outcomes of experiments, it's confirmed that the IWOA-CNN has an excellent performance in expression recognition.

The rest of this article is organized as follows: “Related work” section discusses about the work related to the proposed work in detail. “Image pre-processing” section introduces the image pre-processing steps of the pet dog expression dataset. “Network architecture and applied strategies” section provides the detail information about the designed CNN model architecture. “Whale optimization algorithm and improvement strategies” section makes a brief exposition of the basic WOA and strategies applied to improve the basic algorithm. Several experiments and the discussion of their results are described in “Experimental results and discussion” section. This article ends with a conclusion and the future research expectation revealed in “Conclusions” section.

## Related work

A great number of related works have been carried out as the result of facial expression recognition contributes to various applications such as computer vision, image processing and bioinformatics. In the field of facial expression recognition, computers analyze images of faces to understand the creatures' expressions and identify different emotions. Many optimized models and improved algorithms for facial expression recognition have been developed recently, which will be discussed in this section.

Sujata et al.^24^ proposed a modular approach for facial expression recognition using Euler principal component analysis (PCA). This approach reduces the influence of illumination on image content to a certain extent, while PCA-based method suffers from a problem that the projection maximizes the variance of all image data, which has a negative impact on the recognition accuracy. Kola et al.^25^ utilized LBP combined with adaptive window to extract facial image features for recognizing different expressions of a person. LBP utilizes the relationship between a pixel and its surrounding pixels to quantify the pixel. LBP has a good performance on describing objects with obvious texture, however, it is easy to ignore image features with less obvious texture features. Since there may be only slight differences between different expressions in facial expression recognition, LBP is not widely used in facial expression recognition.

In recent years, with the characteristics of simultaneous training and feature extraction, deep learning has received significant attention in the academic community. Deep neural network (DNN) was the first deep learning model which is used for identifying patterns in high-dimensional image data. The main disadvantage of DNN is that it takes a lot of time to consume in the training process and has the problem of over-fitting. CNN is a representative model in deep learning. Through reducing pre-processing, it can improve image, audio and video processing and overcome the shortcomings exposed by DNN. Considering that CNN can continuously combine features in multiple hidden layers to form more abundant and abstract features, it has been incorporated in this work to recognize the facial expressions of pet dogs.

Many naturally inspired algorithms are applied to parameter optimization of machine learning models, and the optimized models are used for image retrieval^26^, state prognostic^27^, voltage monitoring^28^, etc. Parameter optimization scheme is used to seek out the optimal parameters required for model operation to achieve the best operation effect. However, traditional parameter optimization schemes may cost too much time so that their search efficiency is poor. Disadvantage with traditional schemes can be reduced by using swarm intelligence optimization algorithms like Cat Swarm Optimization (CSO), GWO, WOA, SSA, etc. These algorithms constantly seek better parameters by using the information obtained in previous iterations, thus reflecting higher efficiency, which is why they are adopted by many scholars. In addition, there are many strategies used to improve these algorithms to achieve ideal results in a variety of applications. Nadimi-Shahraki et al.^29^ proposed a dimension learning-based hunting search strategy to improve the GWO for solving engineering problems. Yue et al.^30^ utilized Levy flight strategy and lateral inhibition to improve the WOA, and applied it to underwater image matching. Dhabal et al.^31^ deployed differential evolution strategy to improve the SSA for image denoising. In this paper, we proposed an improved WOA based on several improvement strategies to optimize the CNN's operating parameters to achieve facial expression recognition.

## Image pre-processing

This section elaborates how we obtained the dataset for the experiment. Image pre-processing is the initial step in the task of facial expression recognition. Owing to the lack of dataset based on dog facial expression, we collect 315 pet dog images, including 72 of “Normal”, 67 of “Happy”, 58 of “Sad”, 63 of “Angry” and 55 of “Fear”. These images are obtained by taking photos or downloading online, including many breeds of dogs, such as Golden Retriever, Labrador Retriever, Shiba Inu, etc. In order to reduce the interference of other factors, it is significant to detect facial region from collected images. We use the Dlib toolkit to get the image of facial region, the process mainly includes these following steps:

1. Recognize the general region of the dog's face with a dedicated detector.
2. Draw facial contour lines to mark the position of facial features using a face shape predictor.
3. Calculate and mark the key points according to the drawn lines.
4. Transform the recognized region to the facial region to be captured based on the coordinates of the key points.
5. Capture the facial region and convert it to a grayscale image.

**Figure 2:**
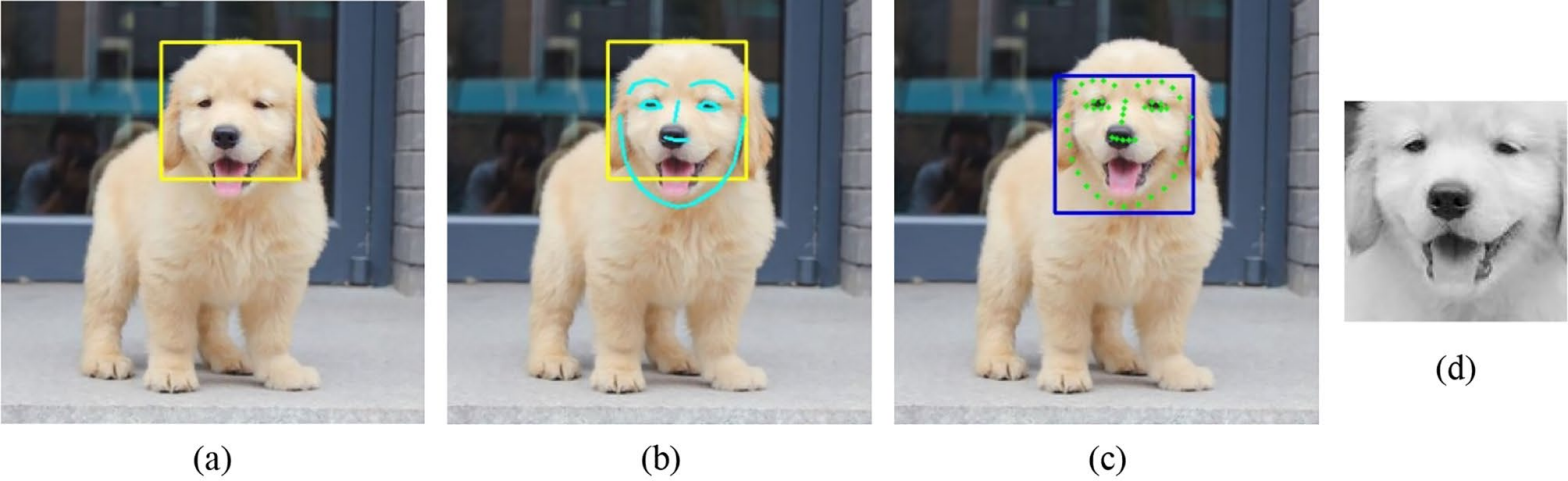
The process of obtaining the facial image. (**a**) Recognize a general region of the dog's face. (**b**) Draw facial contour lines. (**c**) Point out key points of the face and adjust the facial region. (**d**) Capture the facial region and convert it to a grayscale image.

As the reason of many face detection methods are not suitable for dog face detection, we use a dedicated detector based on a trained model, this model is trained from the Columbia Dogs dataset, which was introduced by Liu et al.^32^. It can recognize a general region of the dog's face (as shown in Fig. 2a) and has a high recognition rate in dog face detection. Due to the difference in face shape of different breeds of dogs, the location of the recognized region needs to be adjusted. Consider the different opening degrees of the dog’s mouth in different images, we decide to use a shape predictor to draw facial contour lines (as shown in Fig. 2b) and calculate the key feature points of eyes, nose, and cheeks. Its prediction method is based on the ERT algorithm, this algorithm uses cascaded regression tree to regress the face shape from estimated shape to real shape. It takes about 1 ms to detect the position of facial features. Similar to^33^ and^34^, this cascade regression method is also effective even though feature points are partially missing in the sample image. The iterative algorithm of the regression process can be presented by the following formula:

1 $$\hat{S}^{{\left( {t + 1} \right)}} = \hat{S}^{\left( t \right)} + r_{t} \left( {I,\hat{S}^{\left( t \right)} } \right), t = 1, 2, \ldots , N$$

where *N* is the number of rounds of the regression, ${\widehat{S}}^{(t)}$ is the current estimated shape (i.e., position vectors of the key points) of round *t*, and ${\widehat{S}}^{(t+1)}$ represents the estimated shape of the next round. In each round, the regressor ${r}_{t}$ makes its prediction based on the input image *I* and ${\widehat{S}}^{(t)}$, that is ${r}_{t}\left(I,{\widehat{S}}^{(t)}\right)$. The estimated shape is initialized as the mean shape of the training data, and the update strategy is the Gradient Boosting Decision Tree (GBDT) algorithm^35^. This algorithm splits the feature region into some sub-regions, for each separate sub-region, trains a weak classifier to predict the feature value of that sub-region, then performs several predictions based on the difference between the predicted and actual values, and assign different weights to the values of each prediction. Finally, the predicted value of the whole region is the weighted sum of every predicative value.

After the general region of the dog's face is recognized, the key feature points of the face are dotted in real time by the above algorithm (as shown in Fig. 2c), so we can adjust the location of the recognized region according to the coordinates of the feature points. When the facial region is confirmed, it is captured and converted to a grayscale image (as shown in Fig. 2d).

**Figure 3:**
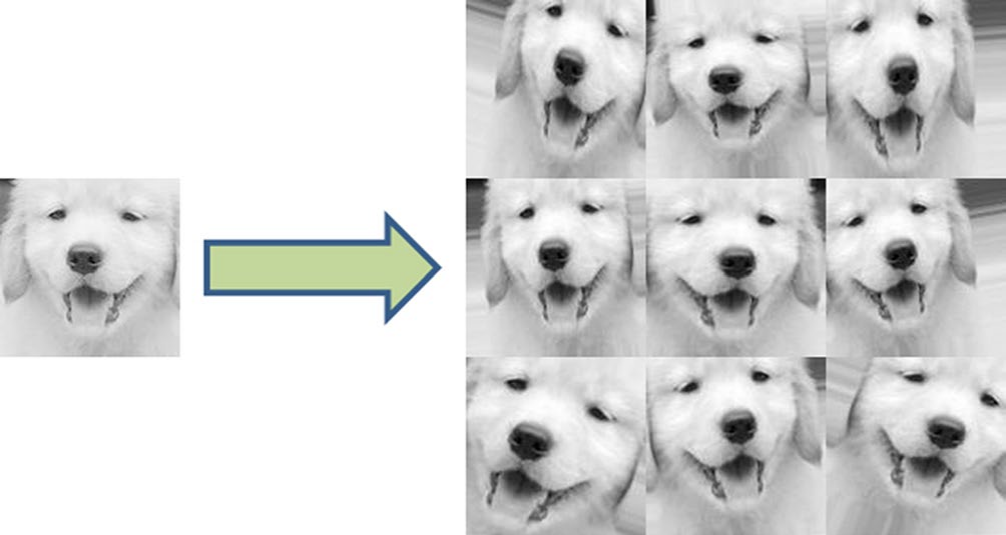
The effect of image data augmentation.

**Figure 4:**
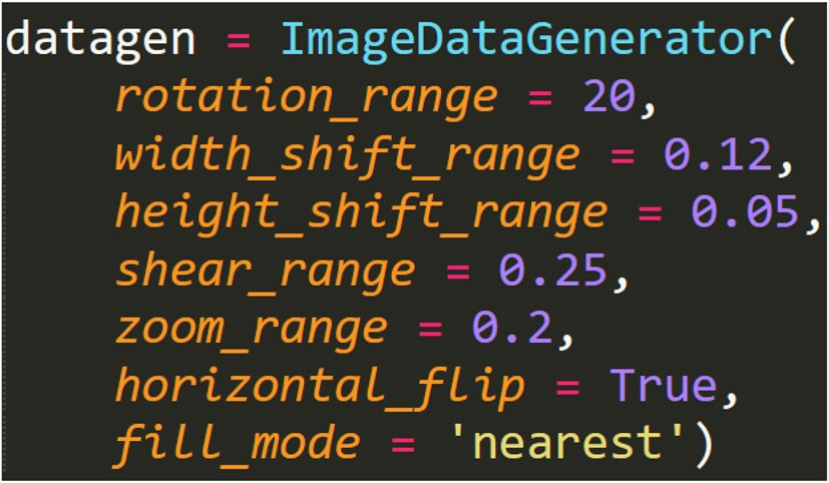
The detailed settings of image augmentation.

As we all know, the operation of the CNN needs a large number data. Considering that the number of dog images we collected is not sufficient, we decide to augment the image data, that is, construct virtual samples to expand the dataset used for model training, methods include the image rotation, translation, zooming and so on. The effect of image data augmentation is shown in Fig. 3. Through image augmentation, the total number of samples in the dataset is expanded to 10 times of the original, and the size of the single image after augmentation is consistent with the original image. The detailed settings of image augmentation are shown in Fig. 4.

## Network architecture and applied strategies

This section will introduce the CNN architecture we designed for facial expression recognition. It is effective in reducing the dimension of the image data and getting the most valuable information present in the input image. Several strategies applied to it will also be explained.

### Network architecture

The network model of deep learning mainly includes CNN, Recurrent Neural Network (RNN), Generative Adversarial Network (GAN), etc. The CNN, which is widely used in speech recognition, image processing, natural language processing and other research fields^36^, it is a specific kind of artificial neural networks for processing data with a similar grid structure, it has three main types of layers, that are: convolutional layer, pooling layer, fully-connected (FC) layer. The convolutional layer is the first layer of the CNN which is used to extract the features in the image^37^. Convolutional layers are usually followed by max-pooling layers or average-pooling layers. The last layer of the CNN (i.e., the output layer) is fully-connected and represents the final output classifications of the network. Different from traditional neural networks, neurons on different layers do not need to be all connected with each other. One of the main advantages of the CNN is that network parameters can be reduced by local perception and weight sharing. When extracting image features, each neuron only perceives local image regions, and then in later layers, the global image information can be obtained by combining these neurons with different local information. In this way, the number of connections can be reduced, in other words, the number of weight parameters that the network needs to train can be reduced. The convolution layer consists of a series of filters which is convolved with the input image independently and transmit its result to the following layer. The convolution operation realizes the dimension reduction of the input image features. The stride in convolution operation represents the process of increasing the step size by which you slide a filter over an input image. The size of pixel matrix which is output by the convolution operation can be calculated using Eq. (2):

2 $$N = \left\lfloor {\frac{W - F + 2P}{S}} \right\rfloor + 1$$

where *W* means the input matrix size is *W* × *W*, *F* means the filter size is *F* × *F*, *P* is the number of padding pixels, *S* represents the stride, *N* means the output matrix size is *N* × *N*. The pooling layer is also called downsampling layer, it is used to reduce the dimension of the pixel matrix and aggregate pixel information, max-pooling layer and average-pooling layer are two kinds of pooling layer, which calculate the maximum value and average value in every sub-region covered by the filter respectively. The pooling layer is usually set as the successor of the convolutional layer, which can help reduce the number of parameters, the computational costs, and prevents over fitting problem.

**Figure 5:**
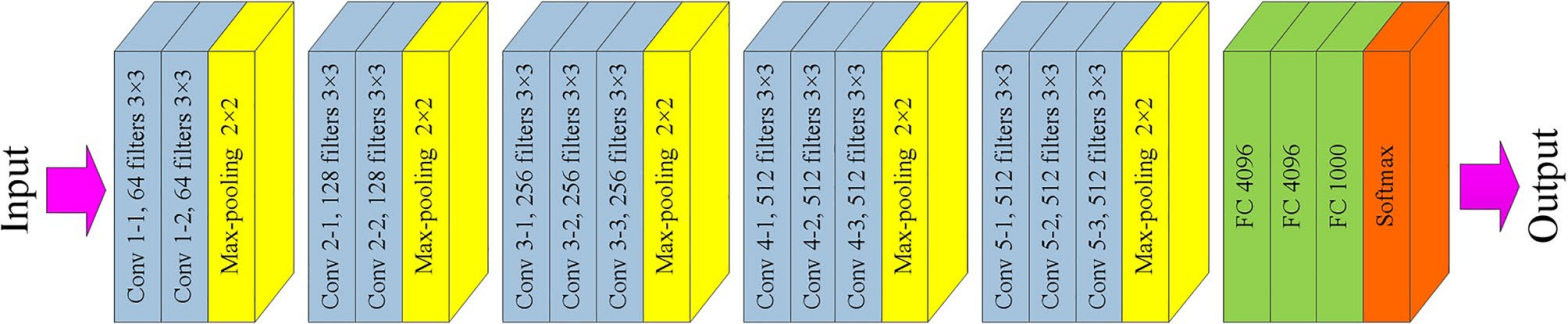
The VGG-16 network architecture.

In this article, the network architecture we designed refers to VGG-16 architecture^38^, which consists of thirteen convolutional layers and three fully-connected layers (as shown in Fig. 5). The ReLU (Rectified Linear Unit) activation function^39^ is assigned to neurons in all convolution layers and fully-connected layer, while the Softmax activation function is assigned to neurons in the last layer to output the classification results. The filter with the size of 3 × 3 is used to expand the number of channels to extract expressive and complex features, and the output data has the same size as the input data through the numeral zero padding. There is a max-pooling layer with a size of 2 × 2 and a step size of 2 behind each continuous convolutional layer to aggregate the transmitted information. With the deepening of the network, the number of filters is also increasing so as to learn more detail information of the input image.

However, since the small size filter is frequently used in the VGG-16 network and the number of filters grows exponentially, this design will lead to a lot of feature maps being output and a lot of storage space being occupied. Through experiments on the VGG-16 model, we find that the first fully-connected layer generates a large number of parameters, which makes the computation huge, the training slow, and consumes more computer resources. Furthermore, for small and medium-sized data samples, using the deep network for training is more likely to cause over fitting problems, which cannot reflect the excellent performance of the deep network. Inspired by AlexNet and GoogleNet^40^, we know that reducing the depth of the neural network to reduce the number of parameters helps prevent over fitting effectively. As the number of convolutional layers is reduced, the large size filters are used to replace the small size filters to maintain the capability of image feature extraction. Therefore, we make the following changes to the VGG-16 model: (1) Replace the first five blocks in the original model with four blocks composed of two convolutional layers and one pooling layer, set the number of filters in the convolutional layer in each block is 32,64,128,256, use 5 × 5 filters on the feature maps of the first two blocks and keep 3 × 3 filters on the feature maps of the last two latter layers. This series of operations can not only reduce the space occupied by feature maps, but also maintain the ability of the model to extract image features. (2) Remove the last fully-connected layer, then reduce the number of nodes in the two remaining fully-connected layers to 512 and 256, and add a random dropout layer after each of them. These dropout layers can set the weight of neurons to zero according to a certain probability (i.e., keep probability) to reduce the coupling between neurons. By the above operations, the number of parameters can be effectively reduced, the model can be prevented from over fitting and its generalization ability can be strengthened.

**Figure 6:**
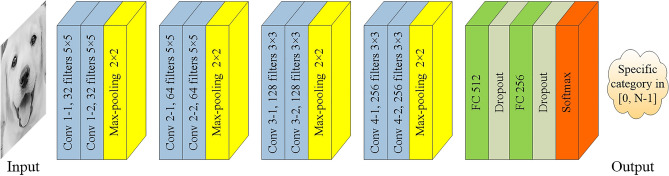
The CNN architecture designed by us.

Now the network architecture designed by us has been determined (as shown in Fig. 6) which consists of eight convolutional layers, four max-pooling layers, two fully-connected layers, two dropout layers and one Softmax layer at last. Every pair of convolutional layer is followed by one max-pooling layer. The dropout layer is the successor of the fully-connected layer and its keep probability is to be optimized. The ReLU activation function is introduced into the neurons in all convolutional layers and fully-connected layers. The Softmax activation function is used in the last layer, it maps the output of multiple neurons to the [0, N − 1] interval to achieve the output of classification results, where N is the number of all categories. Therefore, the designed network model can realize the feature extraction of input images and classify them into different categories. This model requires less training time than VGG-16, and can provide several key parameters to be optimized, which is suitable for optimization using swarm intelligence algorithm.

### L2 Regularization

Regularization techniques play a vital role in the development of machine learning models^41,42^. When fitting a model to some training dataset, the regularization is a common method to avoid over fitting. Eliminating over fitting leads to a model makes better predictions. The regularization of deep learning is a strategy that enables a model to generalize better with new inputs, its specific approach is to add a regularization term to the loss function that needs to be minimized. In this way, the value of the model weight is reduced and the complexity of the model is decreased. Suppose that the loss function *J*_*0*_ of the model is represented by the following formula:

3 $$J_{0} = \frac{1}{m}\sum\nolimits_{i = 1}^{m} {L\left( {\hat{y}^{\left( i \right)} ,y^{\left( i \right)} } \right)}$$

where ${\widehat{y}}^{\left(i\right)}$ and ${y}^{\left(i\right)}$ are the prediction category and actual category of each sample respectively, *m* is the number of samples. The L2 regularization defines regularization term as the sum of the squares of the feature weights, the expression after adding it to the loss function is as follows:

4 $$J = J_{0} + \frac{\lambda }{2m}\left\| w \right\|^{2}$$

where *w* is a multi-dimensional weight vector, *λ* is the regularization parameter. According to the gradient descent principle^43^, the update rule of the weight in this formula is as follows:

5 $$w: = w - \alpha \frac{\partial J}{{\partial w}} = w - \alpha \frac{{\partial J_{0} }}{\partial w} - \alpha \frac{\lambda }{m}w = \left( {1 - \alpha \frac{\lambda }{m}} \right)w - \alpha \frac{{\partial J_{0} }}{\partial w}$$

where *α* is the learning rate, the coefficient in front of the weight is $1-\alpha \frac{\lambda }{m}$ in the above formula, since *α* and λ are both positive numbers, so this coefficient is less than 1. It indicates that the weight is reduced after the L2 regularization is applied, which achieves the purpose of reducing the complexity of the model.

**Figure 7:**
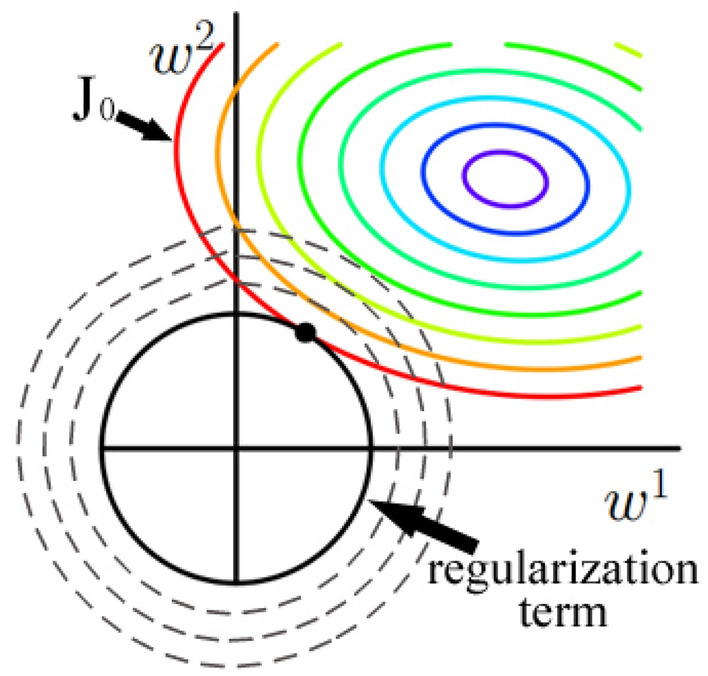
Function graphs of *J*_*0*_ and L2 regularization term.

It can also be comprehended from the geometric perspective, suppose *w* is a two-dimensional weight vector, the function graph of *J*_*0*_ is the color concentric circle located in the upper right part of Fig. 7. If without the L2 regularization, the minimization result of *J*_*0*_ is the innermost purple circle. In this case, the value of *w*^1^ or *w*^2^ may be large. The L2 regularization term's function graph is the black concentric circle located in the lower left part of Fig. 7, when it is added to *J*_*0*_, the optimization target is not only to minimize the value of *J*_*0*_ (i.e., approach the innermost purple circle), but also to minimize the circle representing the regular term. When the circle representing these two functions is tangent, that is, when the vector *w* is located on the black point, the values of *w*^1^ and *w*^2^ are most appropriate. It can be seen that the value of the vector *w* is reduced after the regularization term is added.

In this article, we apply the L2 regularization to the fully-connected layer which has a large number of parameters, so as to achieve the goal of reducing the weight and complexity of the network model. The value of regularization parameter λ is set between 0.0005 and 0.01, and then the intelligent optimization algorithm will be used to search for the optimal value of this parameter.

### Dynamic learning rate

**Figure 8:**
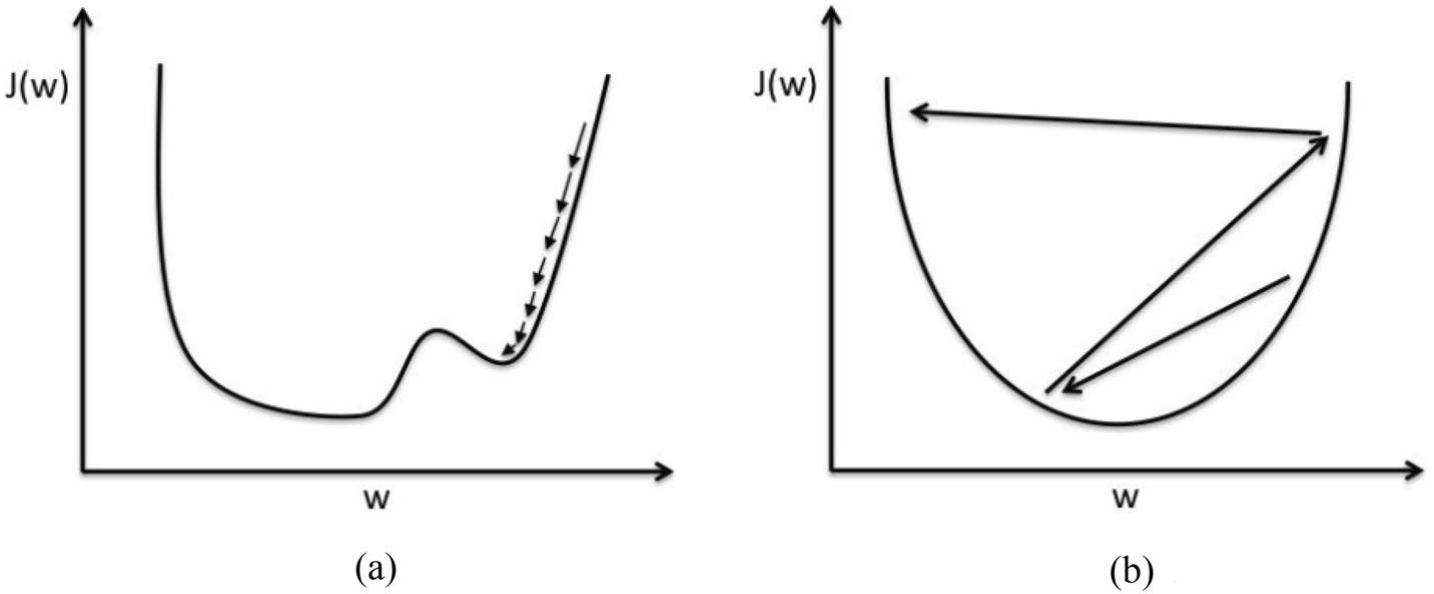
(**a**) Small learning rate: Slow convergence and trapping in local minima. (**b**) Large learning rate: Overshooting.

In deep learning, when training the model, it is necessary to set a learning rate *α* to update the parameters, such as $w:=w-\alpha \cdot \nabla w$. The learning rate is a super parameter that guides us how to adjust the network model weight through the gradient of loss function. It is difficult and important to find a suitable learning rate^44^, if the learning rate is too small, it will lead to slow convergence of training and trapping in local minima (as shown in Fig. 8a), if the learning rate is too large, the loss value will oscillate around the optimal point or even cannot converge (as shown in Fig. 8b).

Therefore, we decide to implement a learning rate decay^45^, that is, set a large learning rate at the beginning of training, and gradually reduce the learning rate as the training process proceeds. The advantage of this approach is that a large initial learning rate makes it easy for the model to escape the local minimum and speed up training. When the global optimum is found later, the problem of oscillation around the optimum is avoided by reducing the learning rate. On the other hand, from the perspective of machine learning, it can be explained that the initial large learning rate inhibits the memory of noise data, while reducing the learning rate is conducive to the learning of complex patterns. Specifically, we perform exponential decay on the learning rate. In the case of exponential decay, the updating formula of the learning rate is shown as follows:

6 $$learning\_rate = initial\_learning\_rate \times decay\_rate^{{\frac{step}{{decay\_steps}}}}$$

where *step* represents the number of steps that have been trained, and a batch of data sent into the model is denoted as a *step*, *decay_steps* means the decay velocity, each time the *step* increases by *decay_steps*, the initial learning rate will be multiplied by the decay rate once. In this article, we set the *decay_steps* to 10, since it is difficult to determine the optimal value of initial learning rate and decay rate, we set them in the range of [0.01,0.8] and [0.89,0.99] respectively, and then adopt the SIOA to explore their optimal values.

## Whale optimization algorithm and improvement strategies

In this section, we will state the basic whale optimization algorithm, propose the improvement strategies for its shortcomings, and explain how to use it to optimize the network model.

### Whale optimization algorithm

It is well known to us that whale is the largest mammal in the world, it has copious emotions and high intelligence, it uses a unique way of hunting, which is called bubble-net. Inspired by this way of hunting, Mirjalili and Lewis^46^ proposed the whale optimization algorithm, which is a meta-heuristic algorithm that describes the special hunting behavior of humpback whales. Suppose there are *N* whales search for prey in the *D* dimension space, the location of prey corresponds to an optimal solution, the position of the i-th whale can be expressed ${X}_{i}=\left[{x}_{i}^{1},{x}_{i}^{2},\dots ,{x}_{i}^{D}\right], i=\mathrm{1,2},\dots ,N$. Whales communicate in the population to move closer to the prey, and their hunting behaviors can be divided into three states: search for the prey, encircling the prey and attacking the prey using a bubble-net method.

When the humpback whale (i.e., the search agent) searches for the prey (i.e., the best solution), it searches randomly based on the position of each search agent, and its position is updated in this state by using a randomly selected search agent rather than the best search agent. Following that, if {|*A*|≥ 1}, as defined in Eq. (8), then let the search agent move according to a randomly selected whale, its position is updated using Eq. (7):

7 $$X_{i}^{t + 1} = X_{rand}^{t} - A\left| {C \cdot X_{rand}^{t} - X_{i}^{t} } \right|$$

where ${X}_{rand}^{t}$ is a position randomly selected from the current population,${X}_{i}^{t}$ and ${X}_{i}^{t+1}$ is the current position and the updated position of a search agent, *A* and *C* are coefficient vectors, their definitions are presented in Eqs. (8), (9) separately.

8 $$A = 2a \cdot r - a$$

9 $$C = 2r$$

where *r* is a random vector in range [0, 1], and *a* is a control parameter, which decreases linearly from 2 to 0 during the iterations. The reduction of *a* makes the search agent closer to the best search agent at the end of iterations.

When humpback whales encircle the prey during hunting, they consider the current best candidate solution as the best solution and try to approach it. In this state, Eq. (10) is used to update the positions of the other whales to approach the best search agent.

10 $$X_{i}^{t + 1} = X_{best}^{t} - A\left| {C \cdot X_{best}^{t} - X_{i}^{t} } \right|$$

where *t* is the current iteration, ${X}_{best}^{t}$ is the position of the best search agent which represents the current best candidate solution, *A* and *C* are coefficient vectors as shown in Eqs. (8), (9). Besides, |*A*| is less than 1 when the whales encircle the prey.

When humpback whale uses a bubble-net to attack the prey, the bubble-net strategy combines two mechanisms. Let us analysis the mathematical model of each mechanism in order to better understand this state.

#### Shrinking encircling mechanism

**Figure 9:**
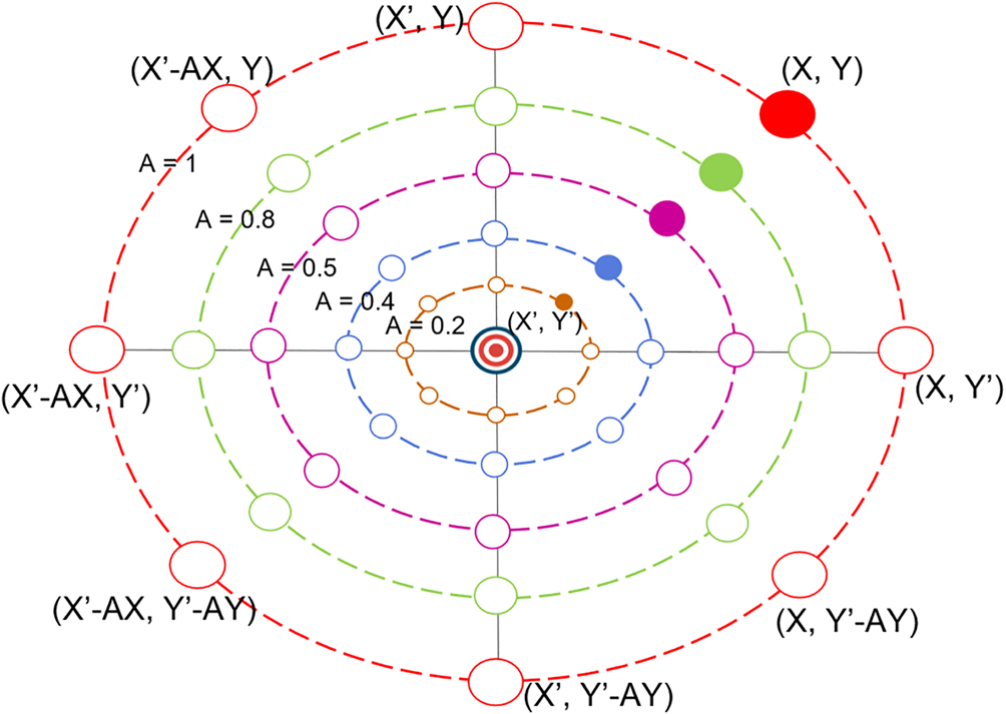
The changes of search agent position under the shrinking encircling mechanism.

The value of *A* in this mechanism is a random value within the [− *a*, *a*] interval, and the value of *a* decreases from 2 to 0 over the course of iterations, as shown in Eq. (8). Assume random values for *A* in range [− 1, 1], so that the new position of a search agent can be anywhere between the current best agent’s position and the agent’s original position as shown in Fig. 9, which illustrates the search agent can move from (X, Y) or other coordinate points to (X′, Y′).

#### Spiral updating position mechanism

**Figure 10:**
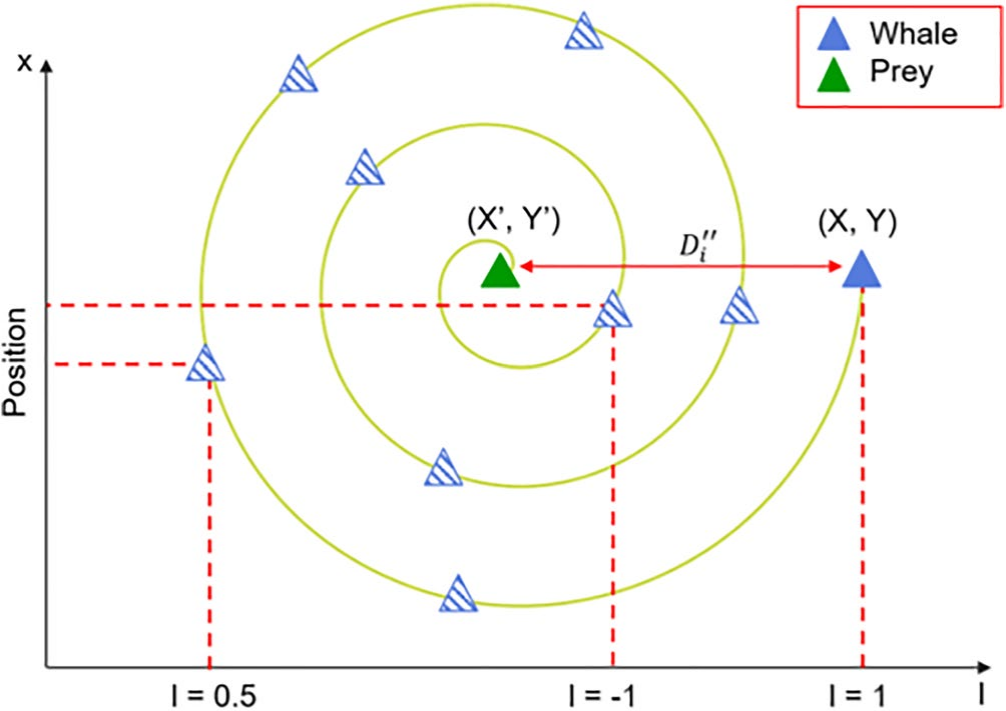
The spiral updating position.

This mechanism starts with the calculation of the distance between the whale (X, Y) and the prey (X′, Y′), then uses Eq. (11) to define the process of a humpback whale moving in a spiral shape while spitting bubbles.

11 $$X_{i}^{t + 1} = D^{\prime} \cdot e^{bl} \cdot \cos \left( {2\pi l} \right) + X_{best}^{t}$$

where ${D}^{^{\prime}}=\left|{X}_{best}^{t}-{X}_{i}^{t}\right|$ represents the distance between the whale and the prey (i.e., the best solution obtained so far), *l* is a random number in range [− 1, 1], and *b* is a constant defining the shape of the logarithmic spiral. Now we can get the mathematical model to describe the humpback whale’s swimming style around the prey using a shrinking circle and also following a spiral-shaped path at the same time, it is represented as Eq. (12).

12 $$X_{i}^{t + 1} = \left\{ {\begin{array}{*{20}l} {X_{best}^{t} - A\left| {C \cdot X_{best}^{t} - X_{i}^{t} } \right|, } \hfill & { p < 0.5} \hfill \\ {D^{\prime} \cdot e^{bl} \cdot \cos \left( {2\pi l} \right) + X_{best}^{t} ,} \hfill & { p \ge 0.5} \hfill \\ \end{array} } \right.$$

where *p* is a random number in range [0, 1], which explains the probability of selecting one of these two methods to update the position of search agents. In other words, the probability of choosing between the two approaches is 50%. Figure 10 illustrates the spiral updating position in detail, where the label of the x-axis is the parameter $l$ in the Eq. (11), and the label of the y-axis means the updated two-dimensional position coordinate.

The whole process of the basic WOA can be described by the following pseudo-code 1.
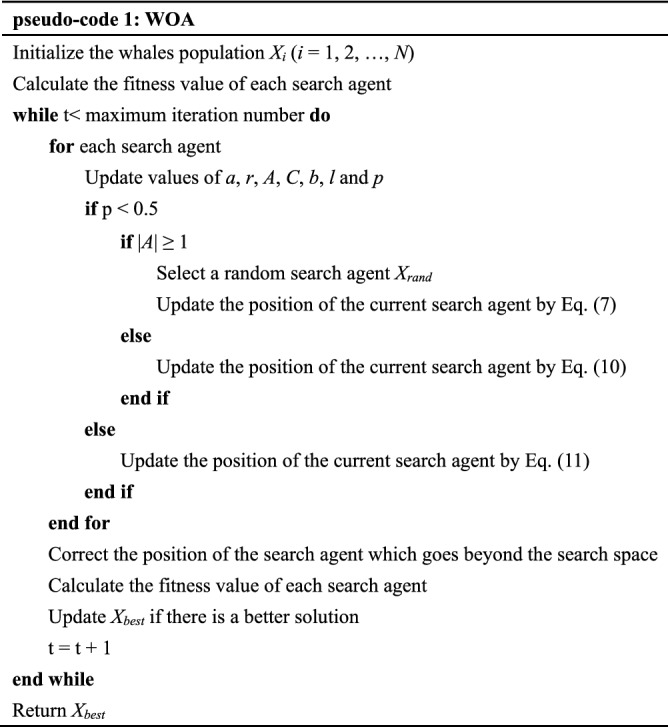


### Proposed improvement strategy

The basic WOA has several shortcomings like low precision, slow convergence speed and easy to trap in the local optimum. In order to overcome these shortcomings, we propose these following improvement strategies to improve the position update method of the basic WOA and prevent it from trapping in local optimum.

#### Nonlinear convergence factor

Similar to other SIOAs, the basic WOA will also encounter the imbalance between global exploration ability and local exploitation ability in the optimization process. In the basic WOA, the coefficient vector *A* controls whether whales conduct global exploration or local exploitation. When |*A*|≥ 1, whales conduct global exploration, and when |*A*|< 1, whales conduct local exploitation. Due to the linear change of the control parameter *a* in the basic WOA can't adjust the global exploration ability and local exploitation ability well, thus we propose a nonlinear convergence factor to instead of the original control parameter^47^. Its expression is shown as Eq. (13).

13 $$a=2+2\mathrm{cos}\left(\mu \frac{t}{max\_iter}\pi +\varphi \right)$$

where *max_iter* is the maximum number of iterations, *t* is the current number of iterations, *μ* and *φ* are relevant parameters, their values are set to 0.5 and $\frac{\pi }{2}$ respectively.

#### Adaptive weight

The basic WOA is prone to trap in the local optimum and premature convergence in later local exploitation. In an effort to make it jump out of the local optimum promptly, we propose an adaptive weight^48^, whose expression is shown as Eq. (14).

14 $$\omega =1-\frac{{e}^{\frac{t}{\mathrm{max}\_iter}}-1}{e-1}$$

Then, we can make use of it to improve the position update methods in the local exploitation stage. To be specific, Eqs. (10), (11) are converted into the following Eqs. (15), (16), separately.

15 $$X_{i}^{t + 1} = \omega \cdot X_{best}^{t} - A\left| {C \cdot X_{best}^{t} - X_{i}^{t} } \right|$$

16 $$X_{i}^{t + 1} = \omega \cdot X_{best}^{t} + D^{\prime} \cdot e^{bl} \cdot \cos \left( {2\pi l} \right)$$

#### Differential mutation

Maintaining the diversity of the population is conducive to avoiding the population from trapping in the local optimum and accelerating the convergence speed of the algorithm to the global optimum. So we propose the differential mutation strategy^47,49^, when the whale's position is updated, the updated position will be converted by this strategy. If the fitness value calculated with the converted position is better than with the unconverted position, the converted position will be adopted to replace the unconverted position, and its corresponding fitness value will also replace the original. The position conversion method of this strategy is expressed in Eq. (17).

17 $$X_{i}^{t + 1} = r_{1} \cdot \left( {X_{best}^{t} - X_{i}^{t} } \right) + r_{2} \cdot \left( {X_{rand}^{t} - X_{i}^{t} } \right)$$

where *r*_1_ and *r*_2_ are random values in range [0, 1], ${X}_{i}^{t}$ is the updated position, ${X}_{i}^{t+1}$ is the position converted by the differential mutation strategy.

Now we refer to the algorithm that has applied the above improvement strategies as IWOA, and its execution process is illustrated in detail by the following pseudo-code 2.
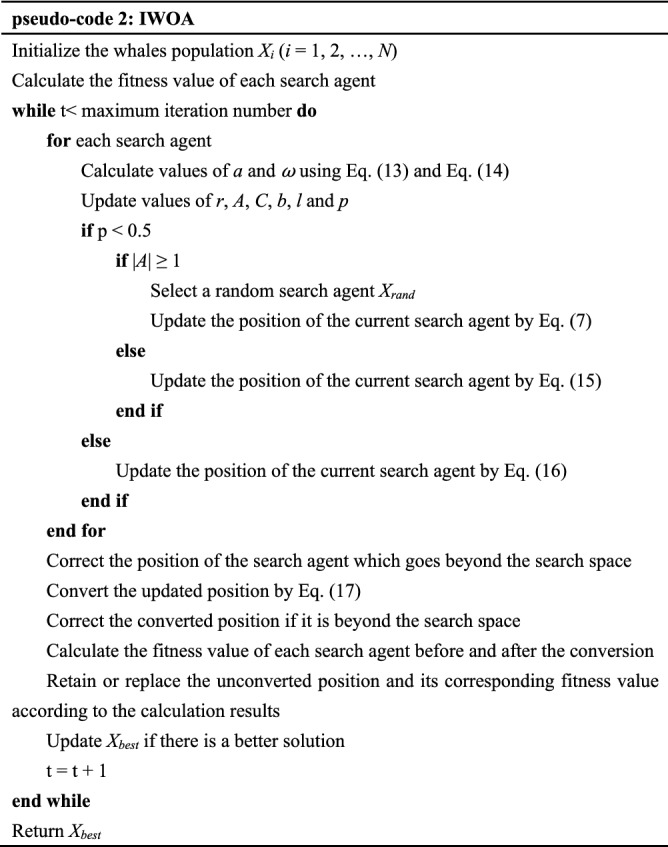


After the IWOA is defined, we will use it and the basic algorithm to optimize several parameters in the CNN model for comparative experiment, these parameters include the keep probability of the random dropout layer, the L2 regularization parameter λ, the initial learning rate and decay rate for setting a dynamic learning rate. The optimization target of the IWOA is to improve the recognition accuracy of the validation set as much as possible.

## Experimental results and discussion

In this section, we will illustrate a series of experimental results to reflect the superiority of using the IWOA, including benchmark function test and facial expression recognition experiment. In the benchmark function test, the basic WOA, IWOA and other relevant intelligent optimization algorithms will be utilized to search the optimal solution of several benchmark functions. In the facial expression recognition experiment, we employ a variety of classifiers for comparative experiments, including Support Vector Machine (SVM), LeNet-5^50^, unoptimized CNN, CNN optimized by the basic WOA (WOA-CNN), CNN optimized by the IWOA (IWOA-CNN). They are applied not only to the dog expression recognition, but also to the human expression recognition based on several ready-made datasets.

### Benchmark functions test

We adopt five kinds of intelligent optimization algorithms in this test, PSO^51^, GWO^52^, SSA^53^, basic WOA and IWOA are included. They are utilized to search the optimal solution of eight distinct benchmark functions. The unimodal functions of the eight functions are shown in Table 1, and the multimodal functions are shown in Table 2. To ensure the fairness of this test, keep the following parameters consistent during the experiment: the dimension of each function is set to 30, the maximum iteration number of each algorithm is 500, and the population size is 100. All algorithms are coded in Python, and the experimental platform is a PC with windows 10 operating system, Inter Core i5 CPU @2.60 GHz, GP107 GPU and 16 GB memory space.

**Table 1:** The unimodal benchmark functions.

No.	Function expression	Dim	Range	f_min_
*F*_1_	$\sum\nolimits_{i = 1}^{n} {x_{i}^{2} }$	30	[− 100, 100]	0
*F*_2_	$\sum\nolimits_{i = 1}^{n} {\left| {x_{i} } \right|} + \prod\nolimits_{i = 1}^{n} {\left| {x_{i} } \right|}$	30	[− 10, 10]	0
*F*_3_	$\sum\nolimits_{i = 1}^{n - 1} {\left[ {100\left( {x_{i + 1} - x_{i}^{2} } \right)^{2} + \left( {x_{i} - 1} \right)^{2} } \right]}$	30	[− 30, 30]	0
*F*_4_	$\sum\nolimits_{i = 1}^{n} {ix_{i}^{4} } + random\left[ {0,1} \right)$	30	[− 1.28, 1.28]	0

**Table 2:** The multimodal benchmark functions.

No.	Function expression	Dim	Range	f_min_
*F*_5_	$\sum\nolimits_{i = 1}^{n} { - x_{i} \sin \left( {\sqrt {\left| {x_{i} } \right|} } \right)}$	30	[− 500, 500]	− 418.98 × *D*
*F*_6_	$\mathop \sum \limits_{i = 1}^{n} \left[ {x_{i}^{2} - 10\cos \left( {2\pi x_{i} } \right) + 10} \right]$	30	[− 5.12, 5.12]	0
*F*_7_	$- 20exp\left( { - 0.2\sqrt {\frac{1}{n}\sum\nolimits_{i = 1}^{n} {x_{i}^{2} } } } \right) - exp\left( {\frac{1}{n}\sum\nolimits_{i = 1}^{n} {\cos \left( {2\pi x_{i} } \right)} } \right) + 20 + e$	30	[− 32, 32]	0
*F*_8_	$\frac{1}{4000}\sum\nolimits_{i = 1}^{n} {x_{i}^{2} } - \prod\nolimits_{i = 1}^{n} {\cos \left( {\frac{{x_{i} }}{\sqrt i }} \right) + 1}$	30	[− 600, 600]	0

**Figure 11:**
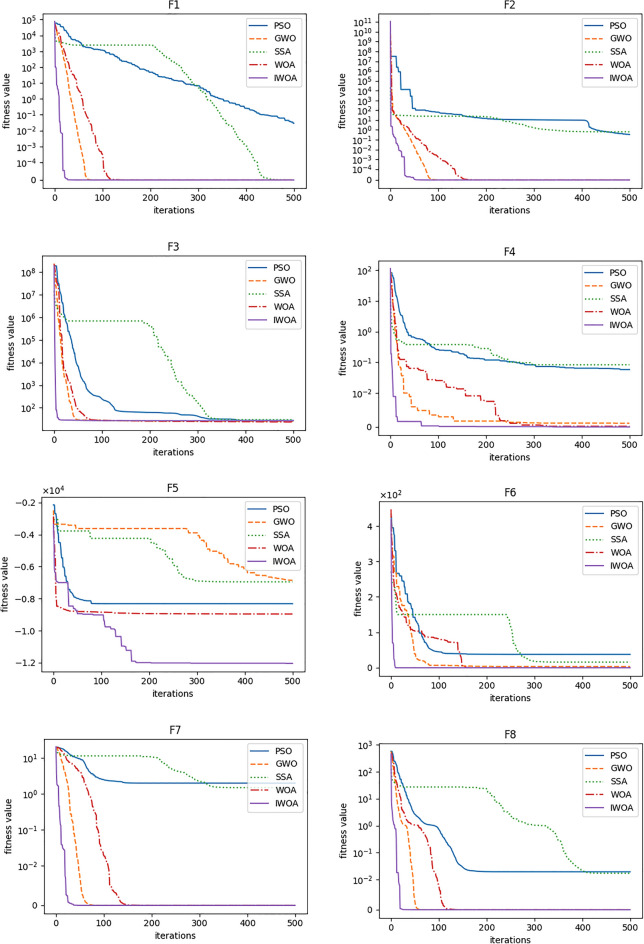
The convergence curves of benchmark functions.

For the sake of reflect these algorithms' performance figuratively, the convergence curve is used to describe the process of searching for function optimal solution. As shown in Fig. 11, the processes of searching the optimal solution by the five algorithms are compared. It proves that IWOA has the fastest convergence speed, and the iterative results are also the best among the five algorithms. Especially in the exploration of *F*_5_, the final fitness value obtained is significantly better than other algorithms. Under the combined action of the nonlinear convergence factor and the adaptive weight, the convergence speed of IWOA to the global optimal solution is accelerated. The differential mutation strategy implemented for the population effectively increases the diversity of the population and helps the algorithm jump out of the local optimum in time, which is obviously reflected in the convergence curve of *F*_4_. It is worth noting that although GWO can find better solutions than WOA in many cases, but the time consumption and parameters of WOA are less than GWO, and these two algorithms are both proposed by Mirjalili et al., while WOA is proposed later. Hence, WOA is a more worthy algorithm to study. In a word, this experiment indicates the superiority of using IWOA to search the optimal solution of function. Therefore, IWOA is an excellent algorithm for solving optimization problems.

**Figure 12:**
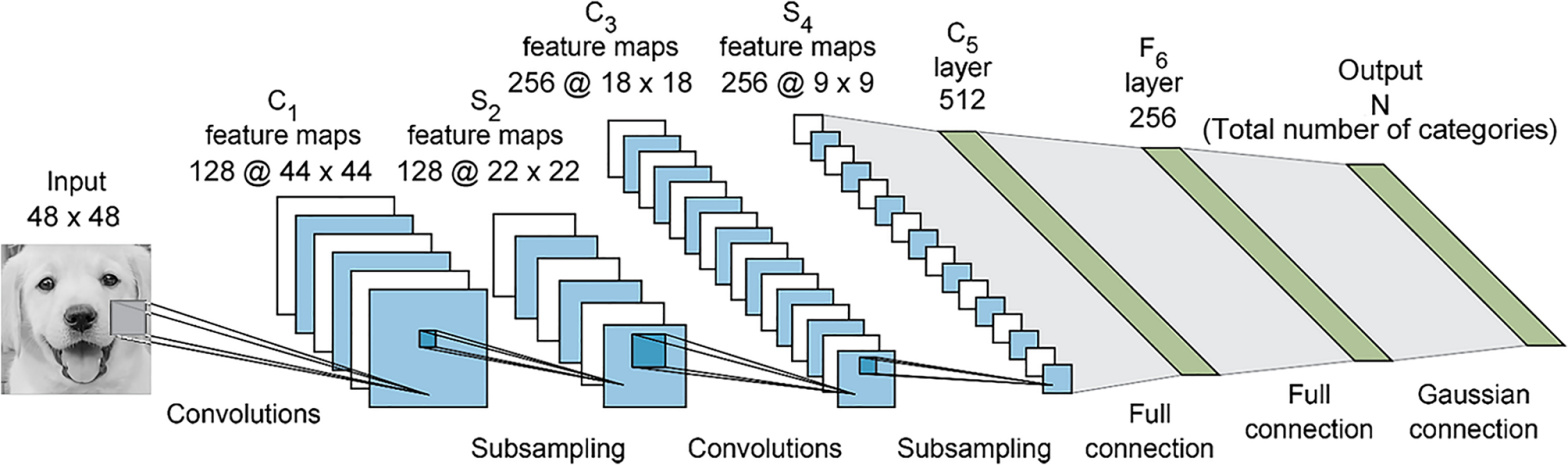
The architecture of LeNet-5 network.

### Facial expression recognition experiment

We have collected 315 images of pet dogs. After the image preprocessing mentioned in “Image pre-processing” section, these images are cropped to 48 × 48 pixels, and we obtain the dataset of dog's facial expression, which contains 3150 images classified into five different expressions (normal, happy, sad, angry and fear). The dataset is divided into two parts: training set and validation set, in which the validation set accounts for 20%. Then, different classifiers are applied to classify these expression images, including SVM, LeNet-5, CNN, WOA-CNN and IWOA-CNN, their parameter settings and architectures are described in Table 3.

**Table 3:** Parameter settings and architecture of classifiers.

Classifier	Parameter settings and architecture
SVM	penalty parameter *C* = 10, kernel parameter γ = 0.1
LeNet-5	architecture is shown in Fig. 12, the ReLU activation function is applied to the convolutional layers and the fully-connected layers, the Softmax activation function is applied to the output layer
CNN	keep probability of dropout layers = 0.6, regularization parameter λ = 0.01, initial learning rate = 0.3, decay rate = 0.96
WOA-CNN, IWOA-CNN	keep probability of dropout layers in range [0.2, 0.9], regularization parameter λ in range [0.0005, 0.01], initial learning rate in range [0.01, 0.8], decay rate in range [0.89, 0.99]
All CNN models	architecture is shown in Fig. 6

**Figure 13:**
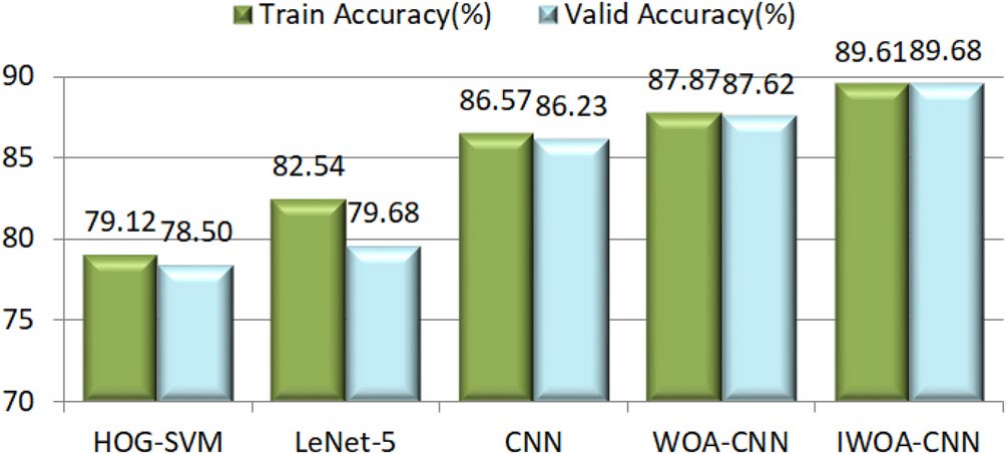
The accuracy of all classifiers.

**Figure 14:**
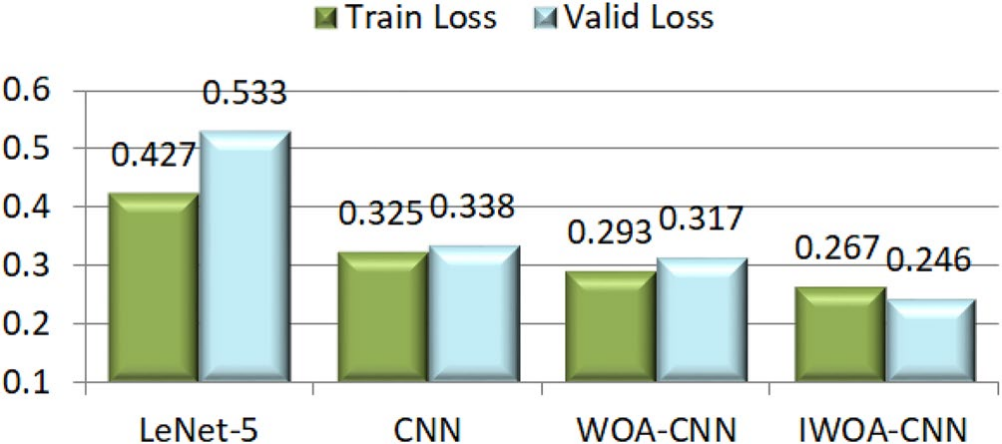
The training losses of all network models.

Among these classifiers, SVM does not belong to the neural network model and it does not have the function of image feature extraction. Thus, the histogram of oriented gradients (HOG)^54^ is utilized to solve this problem, after using it to extract image feature, SVM is utilized to classify these features. Here we refer to this image classification method as HOG–SVM. The categorical cross entropy function is used as the loss function by these network models in the above classifiers, and SVM uses hinge loss function to assess the classification accuracy of all categories. We let the network model train the image data for 200 epochs. In this case, the recognition accuracy and loss obtained from the experiment will tend to converge. We take the accuracy, loss and confusion matrix of expression recognition as the evaluation metrics of the experimental results. The accuracy of expression recognition includes the accuracy of training set and validation set, it is also called recognition rate and calculated by the following formula:

18 $$Recognition \,Rate = \frac{TP}{{TP + FN}}$$

where *TP* and *FN* indicate the number of true positive cases and false negative cases in the evaluation results, respectively. The loss represents the error between the predicted value of the sample and its true value. Generally, the smaller the loss, the higher the accuracy. The accuracy of all classifiers in dog facial expression recognition is shown in Fig. 13. Owing to the loss function used by SVM is different from that of other classifiers, so the training losses of all network models in this experiment are presented in Fig. 14.

From the perspective of recognition accuracy, network models can obtain the higher accuracy than the SVM, and using CNN model is better than other methods in this experiment. After introducing the basic WOA to optimize the parameters of this model, its recognition accuracy is not improved very much by the reason of the optimization ability of WOA is not perfect. However, IWOA improved the recognition accuracy of the original model by more than 3 percentage points. It indicates that IWOA can effectively help the model obtain better operating parameters to improve the recognition accuracy.

**Figure 15:**
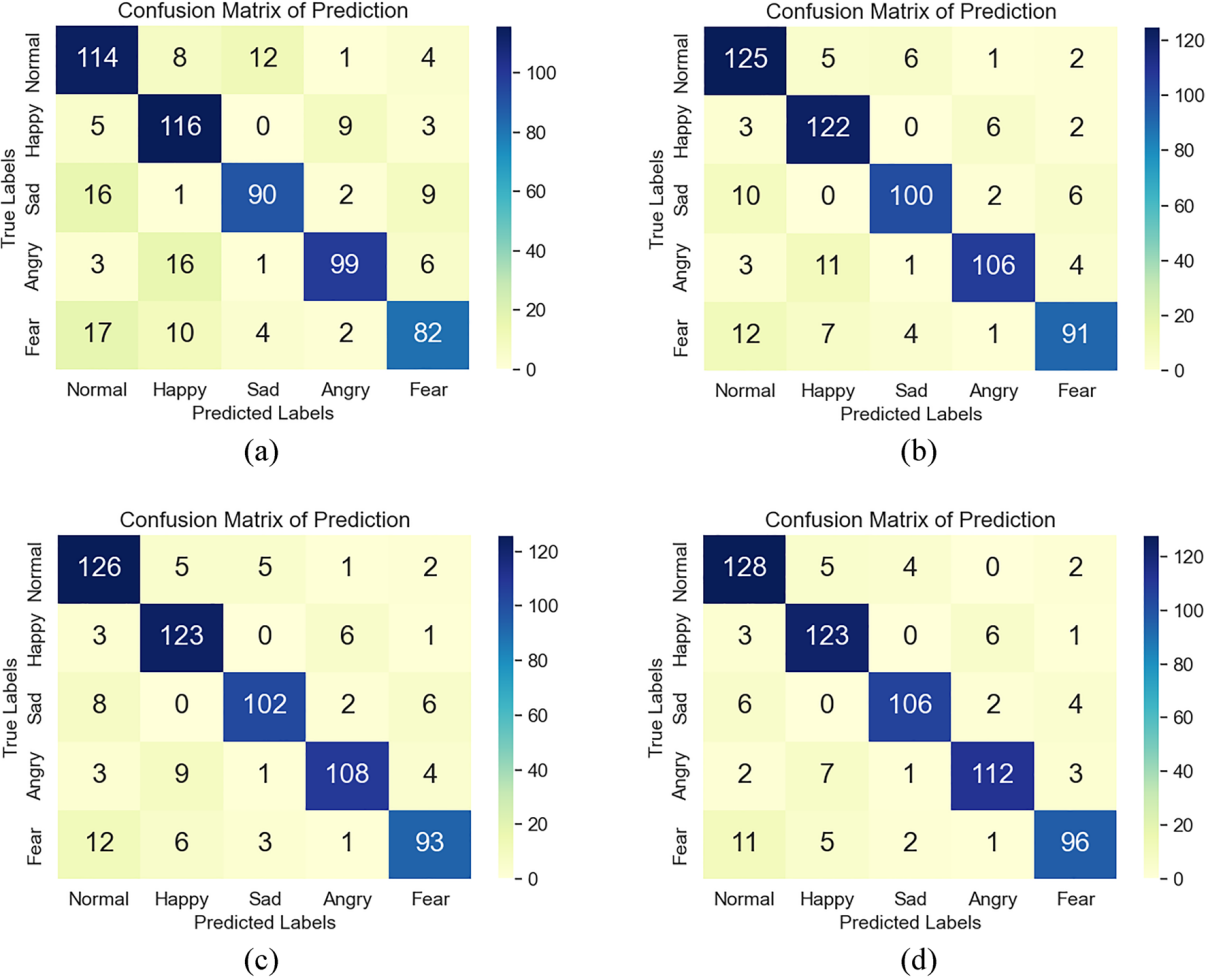
The confusion matrix results of all network models in dog facial expression recognition (generated using Python’s seaborn library). (**a**) The confusion matrix of the LeNet-5. (**b**) The confusion matrix of the CNN. (**c**) The confusion matrix of the WOA-CNN. (**d**) The confusion matrix of the IWOA-CNN.

The confusion matrix results of these network models in dog facial expression recognition are illustrated in Fig. 15a, b, from which we will be aware of the specific situation of sample classification, that is, the normal and happy facial expression categories can be discriminated with a higher recognition rate, while the recognition accuracy of the sad and fear categories does not exceed 90%, it may be ameliorated by using a deeper network model.

**Figure 16:**
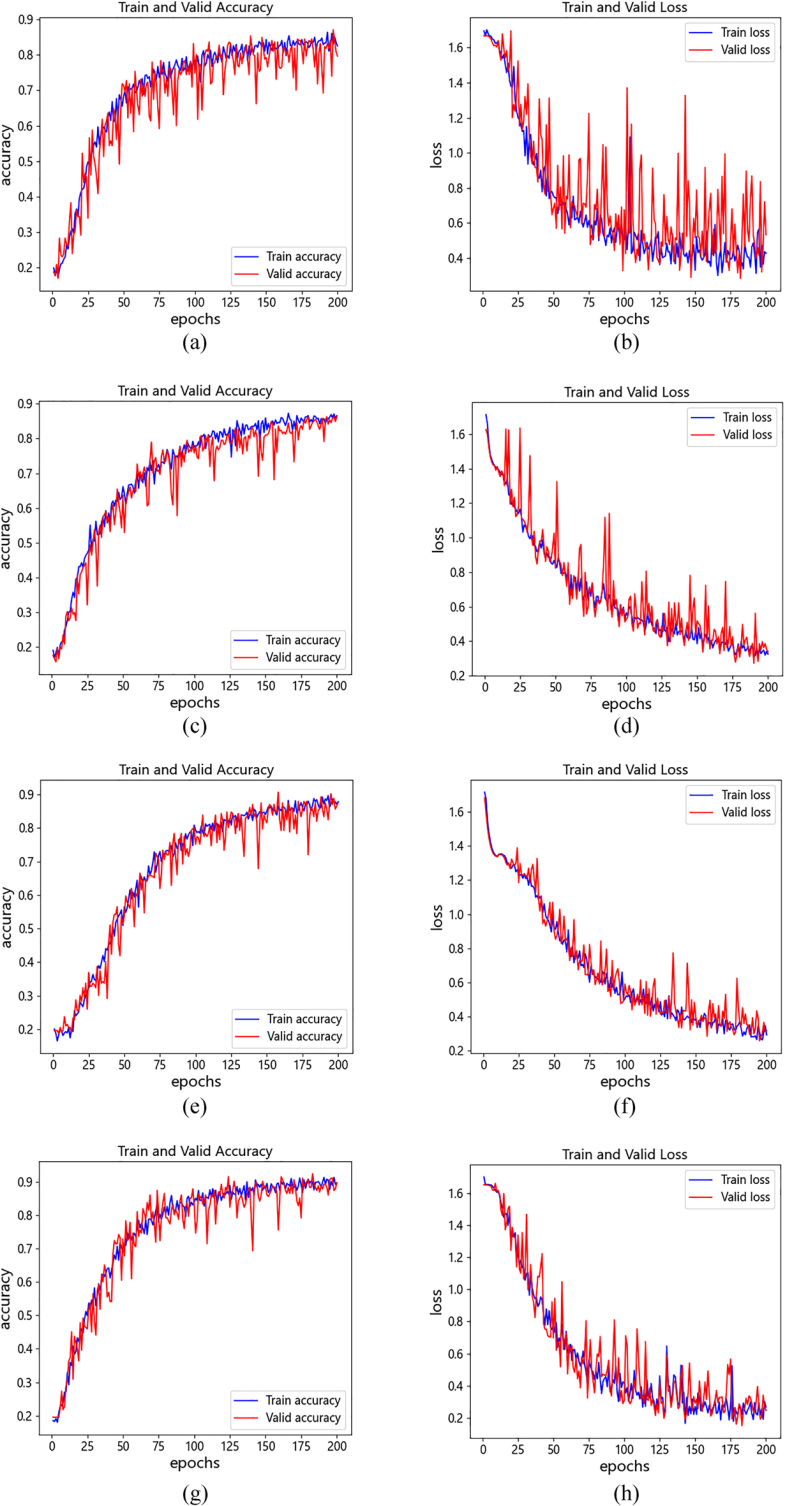
The accuracy curve and loss curve of model training in dog facial expression recognition. (**a**) The accuracy curve of the LeNet-5. (**b**) The loss curve of the LeNet-5. (**c**) The accuracy curve of the CNN. (**d**) The loss curve of the CNN. (**e**) The accuracy curve of the WOA-CNN. (**f**) The loss curve of the WOA-CNN. (**g**) The accuracy curve of the IWOA-CNN. (**h**) The loss curve of the IWOA-CNN.

To observe the process of model training in detail, the accuracy curve and loss curve in the training process of all network models are presented in Fig. 16a, h. These curves illustrate that the LeNet-5, whose architecture is shown in Fig. 12, attain the lowest accuracy of these network models owing to the insufficient number of convolutional layers and without the dropout layer. Moreover, its loss curve is also the most unstable (unable to converge), which indicates that the image features it learned are relatively superficial. However, the CNN model can achieve a higher accuracy, and its loss curve seems more stable, especially after its parameters are optimized by the WOA, and yet the convergence speed of WOA-CNN’s accuracy curve is not significantly faster than that of CNN, result from the parameters optimized by the WOA are still not good enough. Instead, the convergence speed of IWOA-CNN's accuracy curve and loss curve is faster than others, and it can also achieve the highest accuracy and the lowest loss.

**Figure 17:**
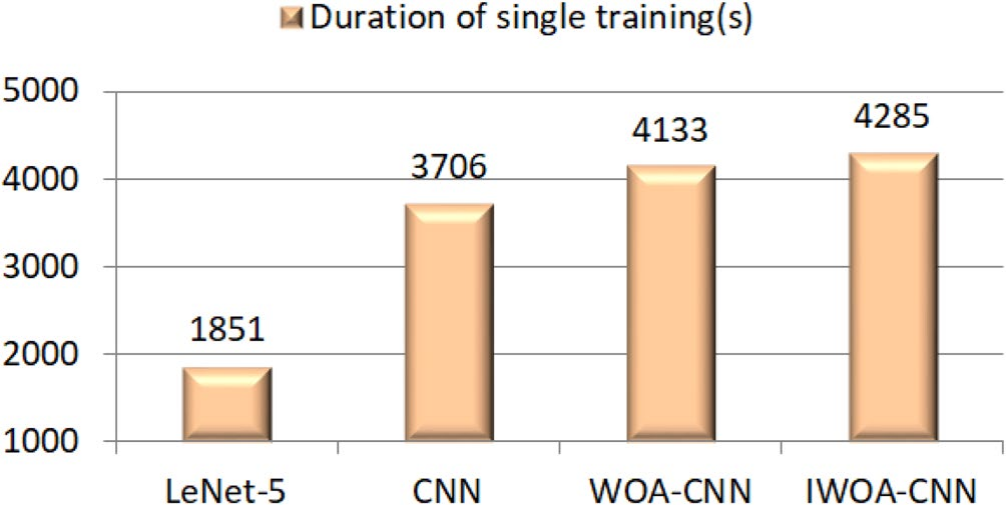
Single training duration of all network models.

**Figure 18:**
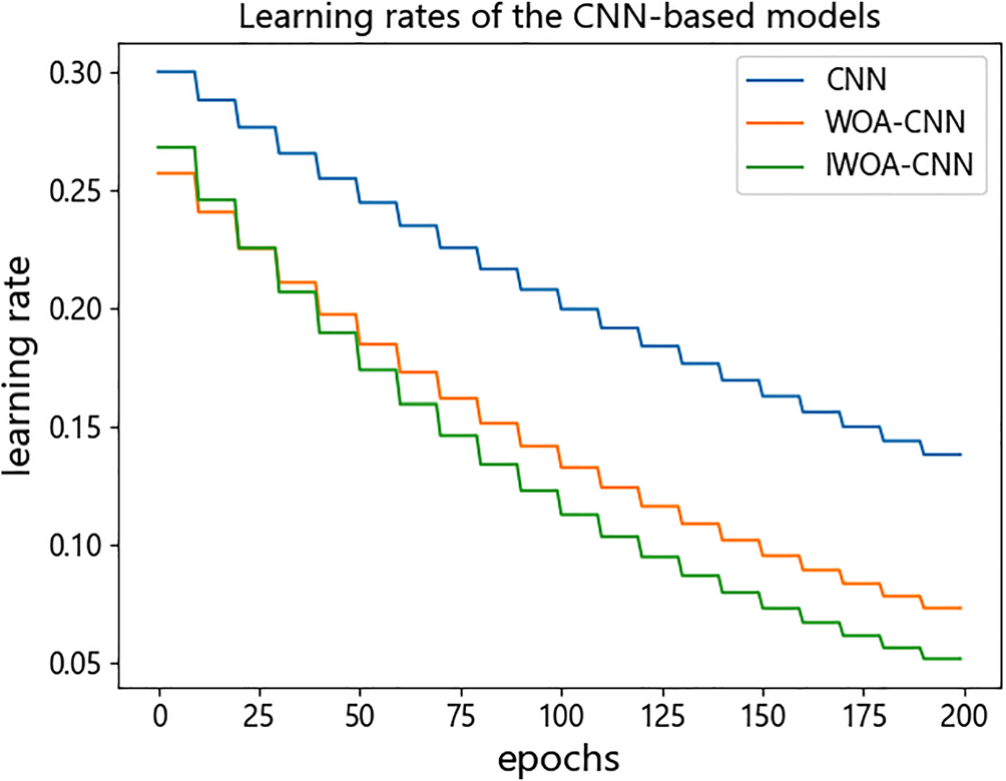
Learning rates of the CNN-based models.

In terms of runtime efficiency, since the process of using HOG-SVM for recognition is to extract image features before model training, which is also different from using network model (the training process includes image feature extraction and recognition), we compared the single training duration of each network model in this experiment. The comparison results are presented in Fig. 17, from which can be seen the LeNet-5 takes the shortest time of these network models due to the simplest model architecture, while the three CNN-based models take a longer time. Of the three, IOWA–CNN takes the longest time, WOA–CNN takes the second place, and CNN takes the shortest time. It is mainly caused by the difference in the number of effective neurons and learning rates at runtime. After WOA optimization, the keep probability of dropout layer in WOA–CNN is about 0.73, and that in IWOA–CNN is about 0.77, which are both higher than that in WOA. Therefore, WOA–CNN and IWOA–CNN need to calculate more neurons than CNN at runtime. By reason of exponential decay on the learning rate, the learning rates of the three CNN-based models will gradually decrease with the training process. The change of the learning rate of the three CNN-based models is shown in Fig. 18, from which we can see that in order to achieve higher recognition accuracy, the learning rate of WOA–CNN and IWOA–CNN in the whole training process is lower than that of CNN. The initial learning rate and decay rate of IWOA–CNN are higher than that of WOA–CNN. On the whole, IWOA–CNN has the lowest learning rate. More neuron calculation and smaller learning rate will lead to an increase in training time, thus forming the difference of training time of each network model, as shown in Fig. 17. Although the optimized model will increase the training time, its improvement of recognition accuracy is also obvious. All things considered, IWOA–CNN has the best performance among all classifiers in this experiment.

Besides, we also have applied these classifiers to the human expression recognition based on several ready-made datasets, like Japanese Female Facial Expressions (JAFFE)^55^, CK+^56^ and Oulu-CASIA NIR&VIS facial expression database (Oulu-CASIA)^57^. JAFFE is a dataset of Japanese women that has 7 kinds of facial expression with 213 images of 256 × 256 pixel resolution. CK+ dataset contains 8 categories of expressions with 593 images of 640 × 490 pixel resolution. Oulu-CASIA contains 2880 image sequences with 6 different facial expressions under 6 different lighting conditions, and we select the last frame of all image sequences in 80 themes under the strong visible light scene (each theme corresponds to 6 different expression image sequences), there are 480 images for the experiment in total. The brief information of these datasets is shown in Table 4. These three datasets are all established in a laboratory environment, but their recognition difficulties are different. Among these datasets, CK+ and JAFFE have better image quality and their recognition accuracy is relatively high in many studies. Moreover, CK+ has more samples than JAFFE, so it is less difficult to recognize. Due to the influence of light, many expression images in Oulu-CASIA are not very clear, so Oulu-CASIA is the most difficult to recognize in these datasets.

**Table 4:** Brief information of several expression datasets.

Name	Categories of expression	Number of images	Resolution
JAFFE	Happiness, sadness, surprise, anger, fear, disgust, neutral	213	256 × 256
CK+	Neutral, sad, surprise, happiness, fear, anger, contempt, disgust	593	640 × 490
Oulu-CASIA	Surprise, happiness, sadness, anger, fear, disgust	2880	320 × 240

**Figure 19:**
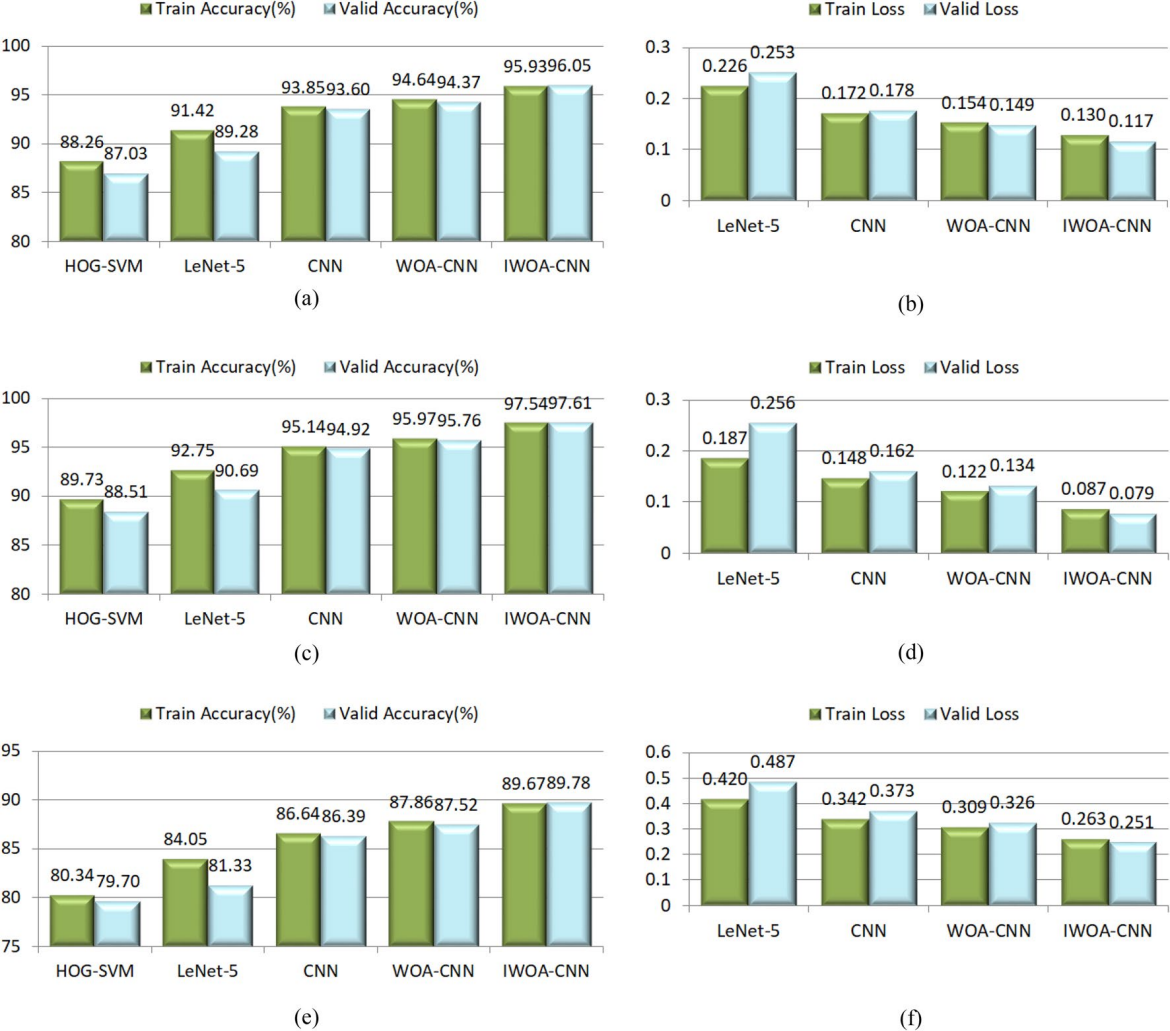
The recognition accuracy and losses on different expression datasets. (**a**) Recognition accuracy on JAFFE. (**b**) Losses on JAFFE. (**c**) Recognition accuracy on CK+. (**d**) Losses on CK+. (**e**) Recognition accuracy on Oulu-CASIA. (**f**) Losses on Oulu-CASIA.

To guarantee the correctness of the experimental results, we capture the facial region in the image and resize it to 48 × 48 pixel, then, the data enhancement technology is used to balance the number of samples of each category, and build virtual samples to expand the total number of samples. For each dataset considered, 80% is treated as the training data, and the other 20% is for validation. Let these classifiers train each dataset for 10 times, and average the results of each training as the final score. The recognition accuracy of all classifiers and the training losses of all network models on these datasets are presented in Fig. 19a–f.

These above experimental results indicate that the recognition accuracy of human facial expressions is higher than that of dogs in most cases, which is due to there is a large number of dogs breeds, and the facial differences between different breeds of dogs are quite large. The recognition accuracy of the CK+ dataset is highest by the reason of the image quality in CK+ dataset is the best and the difference between expressions is obvious. Owing to the influence of light, the recognition rate of Oulu-CASIA dataset is comparatively low. From the performance of each classifier, the recognition accuracy of CNN model is higher than that of SVM, and these network models are utilized to train each dataset for 200 epochs, maybe the training is inadequate for some datasets. Since the WOA is applied to optimize the parameters of the original CNN model, the recognition rate has increased to a certain extent. Thanks to the strong optimization ability of IWOA, the IWOA–CNN attains the highest accuracy and the lowest loss. On the contrary, the recognition accuracy of LetNet-5 is relatively low because of the insufficient network depth, and the lack of measures to prevent over fitting leads to the accuracy and loss of the training set and validation set are quite different. To summarize, IWOA can greatly improve the performance of CNN model to achieve ideal results in facial expression recognition.

### Informed consent statement

All images of pet dogs in this study are used with the permission of the dog's owner, if the dog has an owner.

## Conclusions

Observing facial expressions is an indispensable link in our daily life communication and lots of researches are predominantly carried out in human facial expression recognition. In this article, the facial expression of pet dog is set as the recognition object, the Dlib toolkit is utilized to capture the facial region in the collected dog images, after that divide these facial images into different categories according to their expressions, and data enhancement technology is applied to expand the number of images so as to obtain the expression dataset of dogs.

A redesigned CNN model based on the VGG-16 network model is proposed, moreover, the dropout layer and L2 regularization are introduced into it for preventing over fitting. Besides, an improved WOA is also proposed, which has better solution accuracy and higher convergence speed, it benefit by the application of the nonlinear convergence factor, adaptive weight and differential mutation strategy. The WOA and its improved algorithm are employed to optimize parameters of the CNN model for the facial expression recognition experiment. The experimental results prove that the CNN model optimized by IWOA has outstanding performance in facial expression recognition.

Last but not least, in virtue of the large number of dog breeds, the number of samples selected for the experiment is relatively limited, and more abundant samples are needed to obtain more convincing results. The primary purpose of this article is to demonstrate the role of meta-heuristic algorithms in enhancing the network model performance. In the future, we hope to discover more effective strategies to improve the meta-heuristic algorithm and plumb its application in different fields, such as feature selection, image segmentation, path planning, etc.

## Data Availability

All data that support the findings of this study are available upon request by contact with the corresponding author.
